# Magnetic aerosol drug targeting in lung cancer therapy using permanent magnet

**DOI:** 10.1080/10717544.2018.1561765

**Published:** 2019-02-23

**Authors:** Mohammad K. D. Manshadi, Mahsa Saadat, Mehdi Mohammadi, Reza Kamali, Milad Shamsi, Mozhgan Naseh, Amir Sanati-Nezhad

**Affiliations:** a Department of Mechanical and Manufacturing Engineering, University of Calgary, Calgary, Alberta, Canada;; b Department of Chemical Engineering, College of Engineering, Shahid Bahonar University of Kerman, Kerman, Iran;; c Department of Biological Science, University of Calgary, Calgary, Alberta, Canada;; d Center for Bioengineering Research and Education, University of Calgary, Calgary, Alberta, Canada;; e Department of Mechanical Engineering, Shiraz University, Shiraz, Iran

**Keywords:** Lung cancer, aerosol drug targeting, bronchial tumor, permanents magnet, computational fluid dynamics (CFD)

## Abstract

Primary bronchial cancer accounts for almost 20% of all cancer death worldwide. One of the emerging techniques with tremendous power for lung cancer therapy is magnetic aerosol drug targeting (MADT). The use of a permanent magnet for effective drug delivery in a desired location throughout the lung requires extensive optimization, but it has not been addressed yet. In the present study, the possibility of using a permanent magnet for trapping the particles on a lung tumor is evaluated numerically in the Weibel's model from G0 to G3. The effect of different parameters is considered on the efficiency of particle deposition in a tumor located on a distant position of the lung bronchi and bronchioles. Also, the effective position of the magnetic source, tumor size, and location are the objectives for particle deposition. The results show that a limited particle deposition occurs on the lung branches in passive targeting. However, the incorporation of a permanent magnet next to the tumor enhanced the particle deposition fraction on G2 to up to 49% for the particles of 7 µm diameter. Optimizing the magnet size could also improve the particle deposition fraction by 68%. It was also shown that the utilization of MADT is essential for effective drug delivery to the tumors located on the lower wall of airway branches given the dominance of the air velocity and resultant drag force in this region. The results demonstrated the high competence and necessity of MADT as a noninvasive drug delivery method for lung cancer therapy.

## 1. Introduction

The respiratory tract is the most vulnerable body tissue and affected by diseases such as asthma, chronic obstructive pulmonary disease (COPD), and lung cancer (Magnusson et al., 2011). It is the third most common cancer after breast and prostate cancers, and the first cause of cancer death in the world (Rafiemanesh et al., 2016). The estimated mortality rate of lung and bronchus cancers is above 25% (Siegel et al., 2014, 2016). Lung cancer is classified as small-cell lung cancer (SCLC) and non–small-cell lung cancer (NSCLC) with three histosubtypes according to its pathological tumor (Supporting Information Table S1) (Gridelli et al., 2015; Kalemkerian, 2015). The primary bronchial tumor is the most common lung malignancy.

A large number of researchers have delved into curing methods for lung cancer tumors. The most effective healing methods are surgical treatment, chemotherapy, radiotherapy, and targeted therapy (Qin et al., 2017). Two-thirds of the detected tumors are in the stage where surgery is challenging (Lee et al., 2011). Surgical treatments are possible for early stages of NSCLC but all surgeries have side effects with a risk of mortality during the surgery (Garon et al., 2015). Chemotherapy and radiotherapy may also lead to infection, nausea, mucositis, and diarrhea (Garon et al., 2015). Targeted passive and active drug delivery is particularly used to interfere with cancer cells progression while minimizing the damage to healthy cells (Sawyers, 2004; Mathur et al., 2015; Shamsi et al., 2018a; Soleimani et al., 2018). In the passive method, particles circulate through the body and accumulate in the targeted place depending upon their size, surface properties, and the properties of the targeted tissue (Kleinstreuer & Zhang, 2003; Luo et al., 2007; Modarres et al., 2018). In the active methods, drug particles are pH sensitive (Liu et al., 2014), carry specific ligands (Pastorino et al., 2006), or responds to external stimuli such as ultrasound (Sun et al., 2017) or magnetic fields (Manshadi et al., 2018a,b; Shamsi et al., 2018b). Among active drug delivery methods, magnetic drug targeting (MDT) is one of the successful conservative drug delivery approaches for therapy purposes (Lunnoo & Puangmali, 2015; Pondman et al., 2015; Ostrovski et al., 2016; Russo et al., 2018). Two main MDT approaches could be considered for lung cancer treatment. First, local drug targeting via microvessels where drug particles are administered intravscularly through the nearest feeding blood vessels and captured in the targeted tissue under a magnetic field (Bar et al., 2009). Second, locoregional drug delivery inside airways where drug-aerosol particles are delivered to the tissue via a pulmonary airway tree and captured by the magnetic field (Dames et al., 2007). The concept of using the magnetic field for the manipulation of aerosol particles was proposed by Dikanskii and Kiselev (Dikanskii, 1998; Ally et al., 2005). The first *in vitro* model for lung MADT was developed by Ally et al. (2005) and the first *in vivo* investigation was presented by Dames et al. (2007). Magnetic aerosol drug targeting (MADT) is known as a promising approach for lung cancer therapy (Unterweger et al., 2014). However, there are still several challenges in employing MADT for cancer therapy. A brief overview of the MADT studies and their performance in the lung cancer therapy is presented in Supporting Information Table S2. According to the previous studies, the particles, mainly made of up iron oxide, within the size range of 5 nm to 10 µm were tested to assess their therapeutic performance. The results have demonstrated that MADT was not effective for small particles of down to nanometer scale (Russo et al., 2018). For pulmonary drug delivery, in particular, particle sizes within the range of 1–5 µm diameter were shown to be more effective (Price et al., 2017). Although these carrier particles are degradable and are removed from the body after drug delivery, their poor biocompatibility has limited their clinical utilization (Babu et al., 2013). However, recent studies presented the high biocompatibility of synthetic aerosol particles for cancer therapy (Verma et al., 2013; Stocke et al., 2015; Price et al., 2017).

Although the primary bronchial cancer is the major cause of almost 20% lung cancer mortality worldwide, only a few noninvasive magnetic drug aerosol delivery techniques have been investigated for this lung cancer (Supporting Information Table S2). For instance, the effectiveness of active MADT technique for the delivery of drug aerosols into a lung cancer tumor tissue was demonstrated (Price et al., 2017). Moreover, particle deposition onto the tumors placed within the airflow was shown to be effective using passive drug delivery methods (Kleinstreuer & Zhang, 2003). However, the magnetic-based deposition of aerosol drug particles into a tumor placed within the airway of the human lung employing a permanent magnet has not yet been studied (Supporting Information Figure S1).

This work applies a permanent magnet for MADT to the bronchial cancer tumor, considering the presence of tumor on different bronchial branches from G1 to G3. The deposition of aerosol particles into the lung Weibel model is simulated using CFD methods (Weibel, 1963). The Weibel model, known as a symmetrical tree lung model of a single adult lung cast, is one of the most widely used lung models applied to different *in vitro* and CFD investigations (Chen et al., 2012; Bauer & Brücker, 2015; Janke et al., 2017; Miguel, 2017; Ren et al., 2017; Tena et al., 2017). The magnetic field could be provided by an external magnet source located outside the body or an implanted magnet placed near the target location (Polyak & Friedman, 2009). For instance, Hasenpusch et al. (2012) investigated the capability of placing an external magnet with a minimum distance of 2 mm away from a lung tissue for capturing magnetic particles in an *in vivo* mouse model. Also, the magnet has been directly implanted at the desired location; for instance, Liu et al. (2013) investigated an implant-assisted magnet placed at the desired location for esophageal cancer therapy. Numerical models have been also inspired by the experimental works and modeled MADT systems for drug delivery to the tumor site where permanent magnet was considered attached completely to the desired location (Xie et al., 2010b). Herein, the distance of the magnet from the target site is considered to be 2 mm which is feasible to be implemented internally or externally. For the external magnet configuration, the magnetic field strength needs to be adjusted regarding the distance from the target site to achieve the desired particle deposition to the tumor site (Shapiro et al., 2015; Shamsi et al., 2018). Since the maximal intensity of permanent magnetic field exerted on human tissues should not exceed 0.4 (T) (ICON-IR, 2009) the permanent magnets applied in the present study were chosen such that the magnetic fields intensities generated into the tissue remain below 0.4 (T). Such a magnetic field could be applied with an external source or an implanted magnet. The effects of different parameters, such as magnetic field strength, airway velocity, particle diameter, magnet size, and bronchial cancer tumor location on the efficiency of particle deposition onto the desired locations are studied.

## 2. Materials and methods

### 2.1. Theoretical background

The present study deals with simulating the delivery of drug particles through the lung influenced by an external magnetic field. The Gauss law and Newton's second law are employed to determine airflow velocity, pressure profiles, exerted magnetic field and its resultant forces, and particle movement inside the lung branches. The main equations to determine airflow through the lung branches are continuity and momentum equations. It is assumed that the body is in the resting condition, so airflow is laminar and the abovementioned equations are defined as Equations (1) and (2) (Kamali et al., 2016).

(1) $$\rho \nabla \cdot \left(u\right)=0$$

(2) $$\rho \left(u\cdot \nabla \right)u=-\nabla p+\mu \nabla \cdot \left(\nabla u\right)+F$$

where *ρ* (kg/m^3^) represents air density, ***u*** (m/s) represents the velocity vector, *µ* (Pa.s) is the dynamic viscosity of air, *P* (Pa) is pressure, and ***F*** (N/m^3^) is the sum of external volume forces. The magnetic field around the permanent magnet is obtained from Equation (3).

(3) $$\nabla \cdot \left(\mu_{0}\mu_{r}(H+M)\right)=0$$

where *µ*_0_ represents magnetic permeability of space, *µ*_r_ is relative magnetic permeability, ***H*** is the induced magnetic field, and ***M*** represents magnetization of the magnet. The magnetization is zero for both lung tissue and air. Therefore, Equation (3) is simplified as Equation (4).

(4) $$B=\mu_{0}\mu_{r}H$$

The Newton's second law for particle tracking inside the lung is defined as Equation (5).

(5) $$\frac{d(m_{p}V)}{dt}=\sum F_{t}$$

The left-hand side (LHS) of Equation (5) represents the momentum change of particles (mv), and the right-hand side (RHS) is the sum of acting forces on the particles, including drag and magnetophoretic forces. Brownian motion of the particles is neglected because particle diameter is larger than 50 nm (Lunnoo & Puangmali, 2015). Schiller-Naumann model, which is an accurate model for 1<Re<1000, is to find the drag force on particles (Loth, 2008).

(6) $$F_{d}=\frac{3\mu C_{D}{Re}_{r}}{4\rho_{p}d_{p}^{2}}m_{p}(u-V)$$

(7) $$C_{D}=\frac{24}{\text{Re}}(1+0.15{\text{Re}}_{p}^{0.687})$$

(8) $${\text{Re}}_{p}=\frac{\rho \left|\mathrm{||u}-\right.\left.\mathrm{V||}\right\|d_{p}}{\mu }$$

where *µ* is dynamic viscosity of air, *ρ*_p_ is particle density, *ρ* is air density, Re is the Reynolds number of air flow, *d*_p_ is particle diameter, *m*_p_ is particle mass, ***u*** is air velocity, and ***V*** is particle velocity. Ferroparticles are magnetized in an external magnetic field and attracted to the magnet. The resultant magnetophoretic force is calculated from Equation (9) (Gao et al., 2007).

(9) $$F_{m}=2\pi r_{p}^{3}\mu_{0}\mu_{r}(\frac{\mu_{r,\;p}-\mu_{r}}{\mu_{r,\;p}+2\mu_{r}})\nabla H^{2}$$

where *r*_p_ is particle radius and *µ*_r,p_ is the relative permeability of particles.

Particle-particle interaction is another influential factor in particle delivery systems; however, interparticle interactions are negligible in a dilute solution. In the present study, 2000 particles are introduced into the inlet. Therefore, the particulate inlet volume fraction is lower than 2% (Feng & Kleinstreuer, 2014) and particle-particle interactions can be considered negligible.

### 2.2. Model definition

There are a variety of different numerical methods and software used to simulate drug delivery within tissues (Barisam & Shams, 2016; Akbar et al., 2017; He et al., 2018). Here we use the COMSOL Multiphysics software to simulate MADT. The numerical procedure is validated against experimental and numerical data from the literature.

The generations G0–G3 in the Weibel model (asymmetric triple bifurcation) is used to simulate the parameters of the MADT model. Two thousand ferroparticles are introduced at the inlet at *t* = 0. A 10 s long simulation of MADT assures that the fate of the released particles is determined. Apparently, the nontrapped particles leave the domain at the outlets. Therefore, the fraction of the trapped particles is enumerated after 10 s in all of the simulations unless otherwise mentioned. Furthermore, boundary conditions (BC) and material properties are detailed in Table 1.

**Table 1.** Model dimensions, boundary conditions (BCs), and material properties used for simulating the lung Weibel model.

Branch	Length (cm)	Diameter (cm)
Weibel model dimensions		
G0	L0 = 12	D0 = 1.8
G1	L1 = 4.76	D1 = 1.22
G2	L2 = 1.9	D2 = 0.83
G3	L3 = 0.76	D3 = 0.56
Property	Air	Particle
BCs and material properties		
Density	1.22 (kg/m^3^) (Pourmehran et al. 2016)	5230 (kg/m^3^) (Manshadi et al. 2018c)
Viscosity	1.78 × 10^–5^ (Pa.s) (Pourmehran et al. 2016)	NA
Inlet	15 (l/min) (Zhang et al. 2005; Pourmehran et al. 2016)	Release
Outlet	Pressure outlet	Escape
Wall	No-slip	Stick (Trap) (Pourmehran et al. 2016)
Diameter	NA	1–7 µm
Magnetic permeability	NA	4.1 (Manshadi et al., 2018c)

Particle deposition fraction (PDF) is calculated from Equation (10).

(10) $$\text{PDF}=\frac{\text{Sticked particles to the desired location}}{\text{Totla number of entered particles to the branch}}$$

### 2.3. Validation

**Figure 1.**
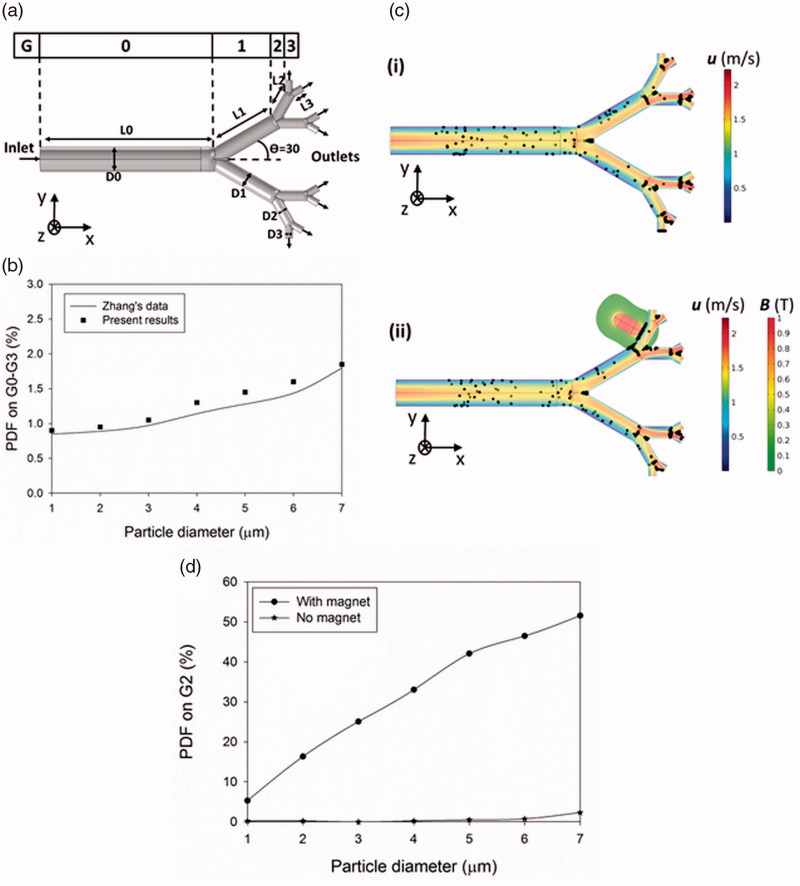
Verification of numerical results of particle tracking and the effect of permanent magnet on particle deposition fraction (PDF). (a) G0–G3 in the Weibel model, (b) comparing numerical results by Zhang's data (Zhang et al., 2005), (c) particle distribution in the lung in the i) absence and ii) presence of external magnetic field 0.5 s after releasing the particles, (d) PDF values on G2 in the presence and absence of the permanent magnet.

To determine the reliability of the numerical model developed in this work, the PDF values of the particles with the diameter of 1–7 µm are compared with Zhang's data (Zhang et al., 2005) with the same boundary conditions (BCs) (Figure 1(a)). Since the numerical method is based on the finite element method (FEM), the grid independency test is performed to find the grid numbers by which the simulation results are independent of the size of elements. The use of above 35,000 number of grids leads to the independency of simulated results from the size of elements for air velocity and particle deposition through the lung. The numerical results of particle deposition fraction were shown to be in a good agreement with the data of previous work (Figure 1(b)). Moreover, the validity of the software for the simulation of magnetic field distribution around a permanent magnet was assessed in our previous study (Manshadi et al., 2018c).

## 3. Results

### 3.1. MADT on lung branches

Following the validation of the numerical model, the effects of the permanent magnet and its resultant magnetic field on particle retention inside the lung in the presence and absence of a tumor is studied. A permanent magnet is employed to generate the magnetic field around the targeted branch in the lung. The profound influence of the magnet inside the body remains below the maximum allowable magnetic field (B ≪ 0.4 (T)) (ICON-IR, 2009). The results show that the permanent magnet has a significant impact on the particle movement through pulmonary airways. The particle trapping on G2 is monitored under no magnetic field and with the magnetic field on the branch (Figure 1(c)). The initial distribution of particles inside the airway flowing in the absence of the magnet leads to the deposition of only a few particles into the G0 wall and bifurcation points, but most of the particles continue their route through the branches with higher velocity magnitude (Figure 1(c), i), (Supporting Information Movie S1). The permanent magnet, however, is shown to deviate effectively the particles path toward the desired place (Figure 1(c), ii). The magnetic field is proven to be effective in increasing particle trapping on the branch where the magnet is located (Figure 1(d)). While the drag force competes with the magnetic force in the MADT for particle manipulation, the magnetic force has a dominant effect on particle movement (Equation (3)). The PDF value increases from 2.3% to 51.5% for particles of 7 µm diameter in the presence of magnetic field. Similarly, the PDF value of 1 µm particles increases from 0.2% to 5.2% on the G2 in the presence of the magnetic field (Figure 1(d)).

**Figure 2.**
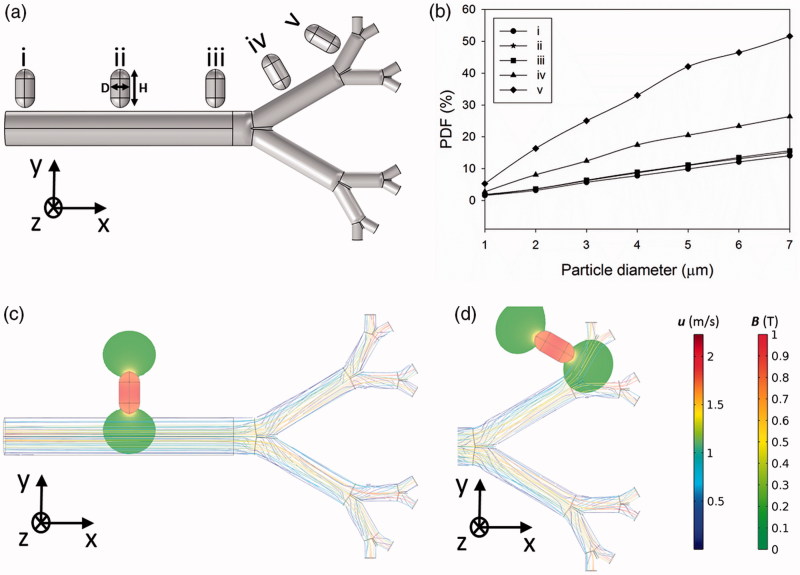
The effect of magnet position on particle retention efficacy. (a) Different magnet locations along the lung airways for a magnet with the size of *H* = 2 cm, *D* = 1 cm). (b) The PDF values at different magnet locations, (c) magnetic flux density for the magnet placed at the position of (ii), (d) magnetic flux density for the magnet placed at the position of (v).

The location of the permanent magnet is also crucial for improving the MADT performance. The magnet is located on different branches to calculate PDFs (Figure 2(a)). Placing the magnet at the smaller branches is more effective on PDF due to the deeper influence of the magnetic field on particles (Figure 2(b)). The magnetic field covers a larger area of the smaller branches (Figure 2(c,d)), therefore most of the particles flowing in smaller branches are trapped within the magnetic field. Since the magnet has a cylindrical geometry, the angle of the magnet with respect to the branches may also affect the PDF in G0. Therefore, this parameter was simulated and the results are shown in Supporting Information Figure S2. The results show that the perpendicular magnet leads to a more profound magnetic field and higher PDFs through the branch respect to the inclined one. Consequently, the magnet is placed perpendicular to the branches in the following simulations.

**Figure 3.**
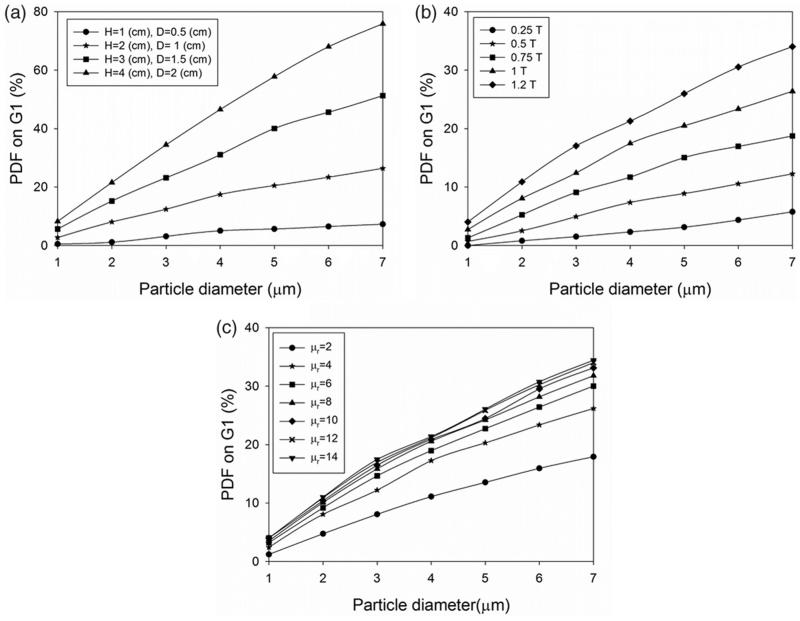
The effects of (a) magnet size, (b) magnetic field strength, and (c) magnetic permeability of the particles on the particle deposition performance in the targeted location.

The magnet size is also shown to be effective in altering the PDF values at different locations along the lung airways branches. The selection of an appropriate magnet size (as large as possible) can provide a higher efficient MADT for the lung, primarily because larger magnets spread the magnetic field in larger areas of the branch, leading to an increase in particle trapping efficacy (Figure 3(a)). For instance, increasing the magnet size from (*H* = 1 cm, *D* = 0.5 cm) to (*H* = 4 cm, *D* = 2 cm) leads to the rise in PDF value from 7.2% to 75.8% for particles of 7 µm in diameter.

The magnetic field strength is also an important parameter in regulating the PDF in different locations along the lung airways branches. For instance, for the situation in which the magnet is placed on G1 (Figure 2(a), iv), and the magnetic field strength is altered within the range of 0.2–1.25 T, the results of PDF values for different particle diameters and magnetic field strength are shown in Figure 3(b). Increasing the magnetic field strength results in higher particle retention at the targeted branch. Also, for the larger particles, the magnetic field strength has a higher impact on particle retention than smaller ones. For instance, increasing the magnetic field strength for a magnet with (*H* = 2 cm, *D* = 1 cm) from 0.25 (T) to 1.25 (T) leads to enhancing the PDF value of 7 µm particles from 5.7% to 34%.

The effect of magnetic permeability of the particles changing within the range of 2–14 on PDF values is further studied. The results show that the increase in the magnetic permeability of particles rises the PDF values in the lung, with a higher rate of increase for low magnetic permeability and a slower rate for the magnetic permeability above 6 (Figure 3(c)).

### 3.2. MADT in a lung with tumor

**Figure 4.**
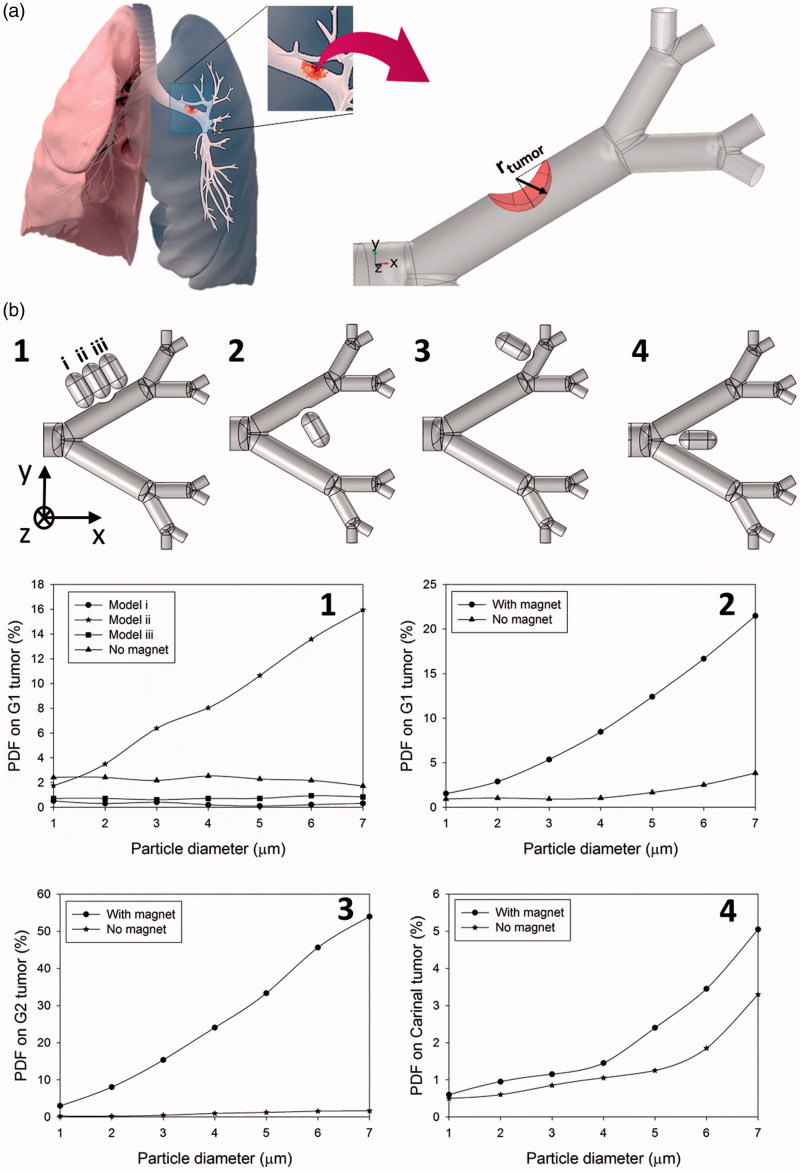
The effect of the permanent magnet on PDFs on lung tumors placed at different locations of the lung airway. (a) Tumor model, (b1) the magnet positions and PDF values for a tumor placed at upper G1, (b2) the PDF for a tumor placed at the lower G1, (b3) the PDF for a tumor placed at the upper wall G2, (b4) the PDF for a tumor placed between G0 and G1 bifurcation point.

In some lung cancers (e.g. squamous cell lung cancer), the tumor starts growing from lung epithelium and extends in some cases until it blocks a portion of the airway (Xi et al., 2015). While some works have modeled the trajectory of particles moving within the branches and their passive deposition on the tumor site (Kleinstreuer & Zhang, 2003; Xi et al., 2015), the active manipulation of particles toward the tumor site has not been investigated. In the present work, we simulate the trajectory of particles when they transport in the airway and investigate the particle deposition fraction (PDF) on the tumor site (surface) under the effect of active magnetic field at the proximity to the tumor site (Figure 4(a)).

First, a tumor with a relative radius of 0.8 *R*_G1_ (*r*_tumor_/*R*_G1_ = 0.8) is considered. The magnet is positioned at three different locations with respect to the upper wall G1 tumor, either before, after or on the tumor site in order to determine the appropriate place for the magnet (Figure 4(b),1). An efficient particle retention occurs on the tumor where the magnet is placed exactly above the tumor which enhances the trapping efficiency of 7 µm particle from 1.7% to 15.9% (Supporting Information Movie S2). Furthermore, when the magnet is located before the tumor site, the particle deposition on the tumor (PDF of 0.31% for 7 µm particles) is even less than the passive targeting (PDF of 1.7% for 7 µm particles). This is mainly because most of the particles are trapped under the magnet and they cannot reach the tumor. Second, the effect of tumor position placed on either the upper or lower wall of the G1 is studied. Comparing Figures 4(b),1 and 4(b),2 shows that the particle deposition is higher on the G1 lower wall tumor (PDF of 21.4% for 7 µm particles) than the upper one (PDF of 15.9% for 7 µm particles). This is primarily because of the higher air velocity near the G1 lower wall causing more particles to move in this region with a higher chance of trapping by the magnetic field. Third, the tumor is placed on G2 (*r*_tumor_/*R*_G2_ = 0.8) as shown in Figure 4(b),3. The magnetic field significantly increases particle deposition enhancing the trapping of 7 µm particles on the tumor from 1.6% to 53.9%. Forth, considering a tumor grown on G0–G1 bifurcation point (Figure 4(b),4), the results demonstrate that although the magnetic field has no significant effect on smaller particles trapping, the larger particles are affected on the tumors growing on bifurcation points.

**Figure 5.**
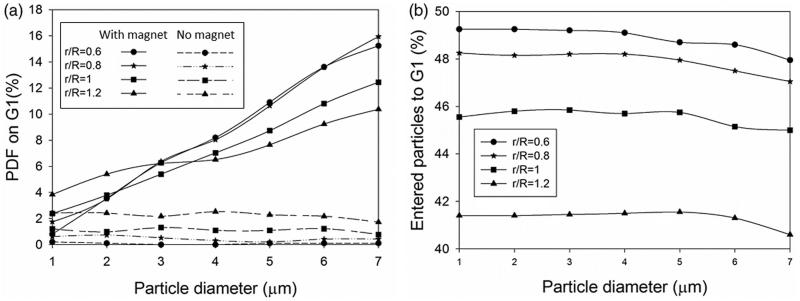
The effects of tumor size on particle trapping. (a) PDF for different G1 tumor size, (b) entered particles to the G1 branch.

Finally, the tumor size was also shown to impact particle deposition. Tumors with four different relative radii *r*_tumor_/*R*_G1_ are considered to study the effect of tumor size on particle retention (Figure 5(a)). Generally, the presence of the magnetic field leads to more particle retention on the tumor surface (especially for larger particles) compared to passive targeting regardless of the size of lesion. Also, increasing the tumor size leads to lowered particle deposition on the tumor (Figure 5(a)). By increasing the tumor size and extending the tumor surface toward the airway, the strength of the magnetic field decreases at the target tumor surface (surface in proximity to the airway). Besides, larger tumors block the branch and decrease the air flow rate. Therefore, less particles flow through the branch as the tumor gets larger which reduces trapping chance of the particles by the magnet (Figure 5(b)).

## 4. Discussion

The application of MADT for lung cancer treatment has been proven using different *in vitro*, *in vivo*, and numerical studies. However, according to the literature of MADT, there is no investigation on magnetic drug delivery to primary bronchial lung cancer tumor a permanent magnet was employed (Supporting Information Table S2). The high potential of employing permanent magnet to enhance drug retention in the region of interest (ROI) of healthy mice was demonstrated in the previous *ex vivo* work (Price et al., 2017). Herein, the permanent magnet capabilities to capture magnetic drug particles on primary human lung cancer tumors for G1–G3 airways are investigated. Several decisive parameters (e.g. the target position, magnet size and position, magnetic field strength, and magnetic permeability of particles) affecting drug delivery to lung tumors with different size and positions were also studied.

Previous studies have shown that for nonuniform magnetic fields, an increase in the distance of the magnetic field source from ROI resulted in a drop in particle deposition (Pourmehran et al., 2016). To deliver magnetic drug particles to the ROI on the first bifurcation, an appropriate distance to the magnetic source needs to be selected (Pourmehran et al., 2015). Using a permanent magnet for drug delivery to the lung tumor, the present study demonstrates that the magnet should be placed exactly above the ROI in order to achieve the maximum drug particle trapping in the targeted location. In addition, according to the Supporting Information Table S2, previous investigations proposed that the presence of magnetic field resulted in higher particle retention in deep lung regions (e.g. alveoli (Dahmani et al., 2009)). Our results also proved that employing magnetic field is necessary to deliver magnetic drug particles to deep lung regions. The presence of a magnetic field is essential in order to overcome the drag force and traps the particles in the ROI. The magnetic field covers a broader area with more profound influence in the smaller branches, therefore PDF remains higher in smaller branches. Besides, magnetic trapping of drug particles is necessary for drug delivery to the tumors especially for the ones growing on the walls of branches. This is mainly because when airflow is divided at the bifurcation points, velocity is not equal in branch sides (Figure 1). Less particles move through the region with low-velocity regions and particle escaping is higher in the high-velocity regions due to the higher momentum. Therefore, PDF is low for the passive drug targeting on the tumors of near wall regions. However, the permanent magnet results in enhancing particle trapping on these tumors in the targeted location (Figure 4). The size and permeability of the particle were also shown to significantly impact the MADT efficiency. It is demonstrated that particle deposition enhances as the particle size increases (Xie et al., 2010a,b). The present results also show that PDF improves by increasing the particle permeability. However, PDF enhancement is insignificant for the relative magnetic permeabilities ranging from 6 to 14.

## 6. Conclusions

This study provides insight into the nature of motion and retention of magnetic particles into the lung airway under an external magnetic field. In the analysis of MDT to lung cancer, the employment of permanent magnet is shown to have a significant effect on particle trapping efficiency with respect to the passive delivery of particles. For tumors that rise on the deeper lung branches where the airflow rate is low, the passive drug delivery has no efficiency and therefore it necessitates the use of magnetic field around the tumor site to capture drug loaded particles. Recommended for lung cancer therapy, MADT could be performed using either an intracorporeal magnet or a magnet placed outside the body.

## Supplementary Material

SI2.wmv

SI1.wmv

SI.pdf
