# Supplementary material for: Magnetic aerosol drug targeting in lung cancer therapy using permanent magnet
Source: Drug Deliv. 2019 Feb 23;26(1):120–8. doi: 10.1080/10717544.2018.1561765 (PMC6394297; doi:10.1080/10717544.2018.1561765)
Supplement: SI.pdf [file IDRD_A_1561765_SM5170.pdf]

Magnetic aerosol drug targeting in lung cancer therapy using permanent magnet

Mohammad K. D. Manshadi, Mahsa Saadat, Mehdi Mohammadi, Reza Kamali, Milad Shamsi, Mozhgan Naseh, Amir Sanati-Nezhad

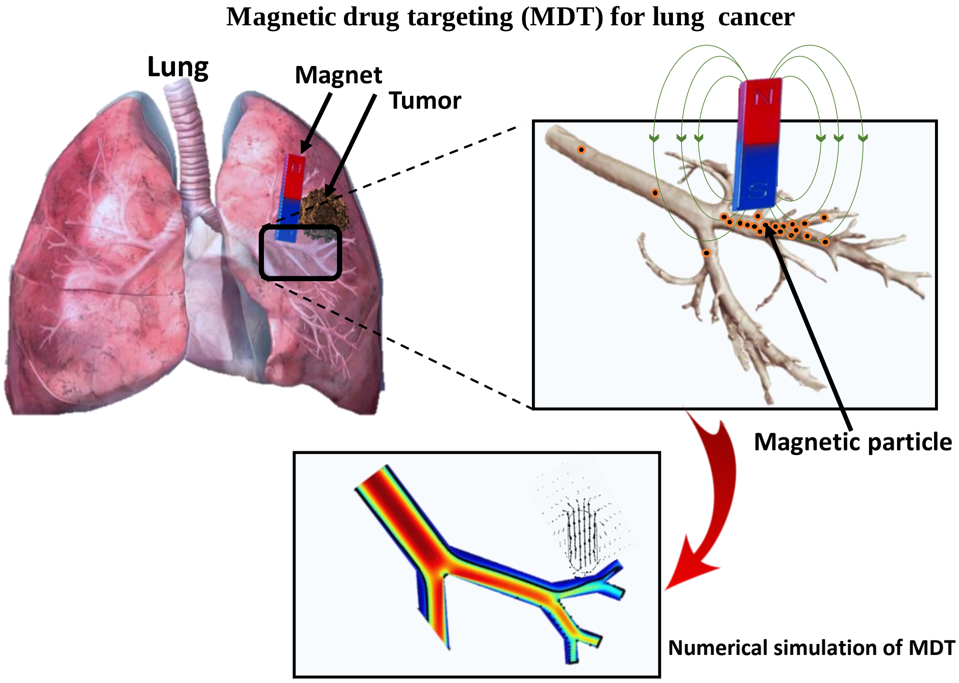

Figure S1. Schematic view of magnetic aerosol drug targeting (MADT) for lung cancer therapy.

Table S1. The development stages of lung cancer

SCLC	**Limited disease:** Disease confined to one hemithorax and can be adequately encompassed by a radiation therapy port (Sekine *et al.*, 2016)	
	Extensive stage: Cancer has proliferated to other regions of the body (Dean and Kreis, 2018)	
NSCLC (Alfonse *et al.*, 2014)	**Occult carcinoma:** Tumor presence is proven by cancer cells detected in sputum. The tumor is not visualized.	
	**Stage 0 (Carcinoma in situ):** The tumor is small and has not spread into the lung.	
	**Stage 1**	**A:** 3 cm or less across the tumor which does not spread in the lymph nodes.
		**B**: Tumor spreads into the underlying lung tissues but does not affect the lymph nodes.
	**Stage 2**	**A:** 3 cm or less across the tumor which spreads in nearest lymph nodes.
		**B:** Several tumors exist and at least one is larger than 5 cm across, or at least one tumor has spread into one of the main blood vessels.
	**Stage 3**	**A:** Tumor spreads in the nearest lymph nodes, or at least one tumor has spread into one of the main blood vessels.
		**B:** Tumor spreads to the larger area of the lung, or lung cancer cell spread into fluid around the lung.
	**Stage 4:** Tumor spreads to another lobe of the lung or any other part of the body.	

Table S2. Brief review on the presented MADT investigations

Study	Authors	Particle property	Procedure	Trapping efficiency	
				Magnet off	Magnet on
In vivo	Dames et al. (Dames *et al.*, 2007)	80 nm SPION*	Wire coil produced a magnetic field on anesthetized healthy mice lung ROI*.	N.A	N.A
	Xie et al. (Xie *et al.*, 2010a)	20 nm Fe_3O_4	Permanent magnet on healthy mice lung ROI*	25%	57%
	Dahmani et al. (Dahmani *et al.*, 2009)	Nanoparticles	Synchronizing the magnetic field activation with the breathing process and delivery of particles into alveoli of animals (Pig)	N.A	N.A
	Hasenpusch et al. (Hasenpusch *et al.*, 2012)	200 nm SPIONs	Permanent magnet on unanesthetized healthy mice lung ROI*	N.A	3-fold higher trapping
	Sadhukha et al. (Sadhukha *et al.*, 2013)	369 nm SPIONs	Magnetic hyperthermia was employed for an orthotopic mouse lung carcinoma tumor model.	N.A	achieved desired doses of SPION
	Verma et al. (Verma *et al.*, 2013)	54.3 nm & 293.4 nm SPION	Biocompatibility of the nanocarriers was evaluated in mice and their therapeutic efficacy was assessed for *in vitro* human lung carcinoma cells.	N.A	N.A
	Price et al. (Price *et al.*, 2017)	3.2 µm NIMs SPION	Permanent magnet was used to deliver dry NIMs to healthy mice lung ROI* and their toxicity was studied in vitro human lung adenocarcinoma cells.	14%	40%
In vitro	Ally et al. (Ally *et al.*, 2005)	1–3 µm carbonyl iron	Permanent magnet on a pipe ROI	10%	48–87%
	Martin et al. (Martin and Finlay, 2008)	high aspect ratio CA* particles in superparamagnetic magnetite	Permanent magnet on ROI over a terminal bronchioles of the human lung model	N.A	Increase by a factor of 1.74
	Xie et al. (Xie *et al.*, 2010b)	0.3-4.5 µm iron oxide	Permanent magnet on a pipe ROI	N.A	100%
	Upadhyay et al. (Upadhyay *et al.*, 2012)	2–4 µm SPIONs	Hyperthermia therapy capability	N.A	100%
	Stocke et al. (Stocke *et al.*, 2015)	Nanoparticles iron oxide	Physicochemical and heating as well as *in vitro* toxicity study on human alveolar epithelial adenocarcinoma	N.A	N.A

			cells were evaluated for hyperthermia therapy.		
	Tewes et al. (Tewes *et al.*, 2014)	Trojan 2.8 µm SPION	Possibility of delivery of Trojan particles to the lung	N.A	N.A
CFD	Xie et al. (Xie *et al.*, 2010b)	0.3-4.5 µm iron oxide	The first study that compares numerical and *In vitro* data of drug delivery on a pipe ROI	N.A	100%
	Martinez et al. (Martinez *et al.*, 2013)	minor diameter of 0.5 µm CA* particles	Deposition efficiency of particles with 6 and 20 aspect ratio in a symmetric bifurcation airway model	N.A	Increase by factors of 1.43 and 3.46
	Pourmehran et al. (Pourmehran *et al.*, 2015)	2-6 µm Doxorubicin	Non-uniform magnetic field used in G0-G2 lung model	12%	100%
	Pourmehran et al. (Pourmehran *et al.*, 2016)	2-6 µm Doxorubicin	Non-uniform magnetic field used in oral cavity, larynx, pharynx and trachea along with six generations of human lung	15%	100%
	Ostrovski et al. (Ostrovski *et al.*, 2016)	0.5-3 µm SPION	Permanent magnet was used on anatomically inspired, space-flling, multigeneration acinar airway tree ROI	40%	100%
	Kenjeres and Tjin (Kenjereš and Tjin, 2017)	0.1-10 µm iron oxide-maghemite core	Magnetic deposition on trachea and last generations in a model from mouth inlet to the eighth generation of the bronchial bifurcations	23%	85%
	Russo et al. (Russo *et al.*, 2017)	5 nm gold/iron-oxide	A coil magnetic source was used in a model of trachea, with its primary and secondary bronchi with target on ROI	50%	47%
SPION: superparamagnetic iron oxide nanoparticles, ROI: region of interest, N.A: not available, CA: cromoglycic acid, NIM: Nano-in-microparticle					

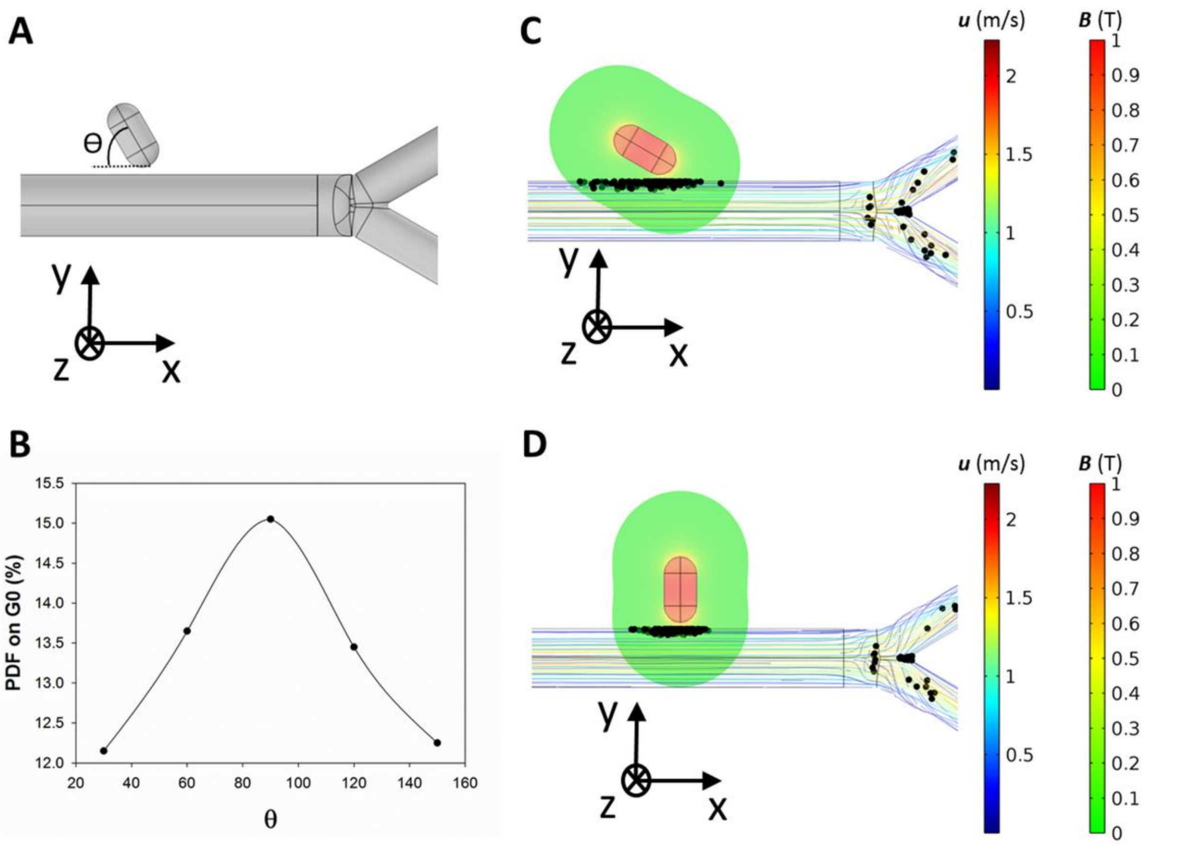

Figure S2. The effect of magnet angle respect to the branches on particle retention efficacy. **A)** Schematic illustration of the inclined magnet, **B)** The PDF values at different magnet's angles, **C)** Magnetic flux density for the magnet placed at ϴ=30 respect to the branch, **D)** Magnetic flux density for the magnet placed at ϴ=90 respect to the branch.

References

Alfonse, M., Aref, M.M. & Salem, A.-B.M., 2014. An ontology-based system for cancer diseases knowledge management. *International Journal of Information Engineering and Electronic Business,* 6**,** 55-63.

Ally, J., Martin, B., Khamesee, M.B., Roa, W. & Amirfazli, A., 2005. Magnetic targeting of aerosol particles for cancer therapy. *Journal of Magnetism and Magnetic Materials,* 293**,** 442-449.

Dahmani, C., Gotz, S., Weyh, T., Renner, R., Rosenecker, M. & Rudolph, C., Year. Respiration triggered magnetic drug targeting in the lungsed.^eds. *Engineering in Medicine and Biology Society, 2009. EMBC 2009. Annual International Conference of the IEEE*IEEE, 5440-5443.

Dames, P., Gleich, B., Flemmer, A., Hajek, K., Seidl, N., Wiekhorst, F., Eberbeck, D., Bittmann, I., Bergemann, C. & Weyh, T., 2007. Targeted delivery of magnetic aerosol droplets to the lung. *Nature nanotechnology,* 2**,** 495.

Dean, A.G. & Kreis, R., 2018. Understanding Lung Cancer: Presentation, Screening, and Treatment Advances. *The Journal for Nurse Practitioners,* 14**,** 316-322.

Hasenpusch, G., Geiger, J., Wagner, K., Mykhaylyk, O., Wiekhorst, F., Trahms, L., Heidsieck, A., Gleich, B., Bergemann, C. & Aneja, M.K., 2012. Magnetized aerosols comprising superparamagnetic iron oxide nanoparticles improve targeted drug and gene delivery to the lung. *Pharmaceutical research,* 29**,** 1308-1318.

Kenjereš, S. & Tjin, J.L., 2017. Numerical simulations of targeted delivery of magnetic drug aerosols in the human upper and central respiratory system: a validation study. *Royal Society Open Science,* 4, 170873.

Martin, A.R. & Finlay, W.H., 2008. Enhanced deposition of high aspect ratio aerosols in small airway bifurcations using magnetic field alignment. *Journal of Aerosol Science,* 39, 679-690.

Martinez, R., Roshchenko, A., Minev, P. & Finlay, W., 2013. Simulation of enhanced deposition due to magnetic field alignment of ellipsoidal particles in a lung bifurcation. *Journal of aerosol medicine and pulmonary drug delivery,* 26, 31-40.

Ostrovski, Y., Hofemeier, P. & Sznitman, J., 2016. Augmenting regional and targeted delivery in the pulmonary acinus using magnetic particles. *International journal of nanomedicine,* 11, 3385.

Pourmehran, O., Gorji, T.B. & Gorji-Bandpy, M., 2016. Magnetic drug targeting through a realistic model of human tracheobronchial airways using computational fluid and particle dynamics. *Biomechanics and modeling in mechanobiology,* 15, 1355-1374.

Pourmehran, O., Rahimi-Gorji, M., Gorji-Bandpy, M. & Gorji, T., 2015. Simulation of magnetic drug targeting through tracheobronchial airways in the presence of an external non-uniform magnetic field using Lagrangian magnetic particle tracking. *Journal of Magnetism and Magnetic Materials,* 393, 380-393.

Price, D.N., Stromberg, L.R., Kunda, N.K. & Muttil, P., 2017. In Vivo Pulmonary Delivery and Magnetic-Targeting of Dry Powder Nano-in-Microparticles. *Molecular pharmaceutics,* 14, 4741-4750.

Russo, F., Boghi, A. & Gori, F., 2017. Numerical Simulation of Magnetic Nano Drug Targeting in Patient-Specific Lower Respiratory Tract. *Journal of Magnetism and Magnetic Materials*.

Sadhukha, T., Wiedmann, T.S. & Panyam, J., 2013. Inhalable magnetic nanoparticles for targeted hyperthermia in lung cancer therapy. *Biomaterials,* 34, 5163-5171.

Sekine, I., Sumi, M., Satouchi, M., Tsujino, K., Nishio, M., Kozuka, T., Niho, S., Nihei, K., Yamamoto, N. & Harada, H., 2016. Feasibility study of chemoradiotherapy followed by amrubicin and cisplatin for limited-disease small cell lung cancer. *Cancer science,* 107, 315-319.

Stocke, N.A., Meenach, S.A., Arnold, S.M., Mansour, H.M. & Hilt, J.Z., 2015. Formulation and characterization of inhalable magnetic nanocomposite microparticles (MnMs) for targeted pulmonary delivery via spray drying. *International journal of pharmaceutics,* 479, 320-328.

Tewes, F., Ehrhardt, C. & Healy, A.M., 2014. Superparamagnetic iron oxide nanoparticles (SPIONs)-loaded Trojan microparticles for targeted aerosol delivery to the lung. *European Journal of Pharmaceutics and Biopharmaceutics,* 86, 98-104.

Upadhyay, D., Scalia, S., Vogel, R., Wheate, N., Salama, R.O., Young, P.M., Traini, D. & Chrzanowski, W., 2012. Magnetised thermo responsive lipid vehicles for targeted and controlled lung drug delivery. *Pharmaceutical research,* 29, 2456-2467.

Verma, N.K., Crosbie-Staunton, K., Satti, A., Gallagher, S., Ryan, K.B., Doody, T., Mcatamney, C., Macloughlin, R., Galvin, P. & Burke, C.S., 2013. Magnetic core-shell nanoparticles for drug delivery by nebulization. *Journal of nanobiotechnology,* 11, 1.

Xie, Y., Longest, P., Xu, Y.H., Wang, J.P. & Wiedmann, T.S., 2010a. In vitro and in vivo lung deposition of coated magnetic aerosol particles. *Journal of pharmaceutical sciences,* 99, 4658-4668.

Xie, Y., Zeng, P., Siegel, R.A., Wiedmann, T.S., Hammer, B.E. & Longest, P.W., 2010b. Magnetic deposition of aerosols composed of aggregated superparamagnetic nanoparticles. *Pharmaceutical research,* 27, 855-865.
